# Culture-based and culture-independent approach for the study of the methanogens and obligate anaerobes from different landfill sites

**DOI:** 10.3389/fmicb.2023.1273037

**Published:** 2024-01-29

**Authors:** Om Prakash, Sahab Ram Dewala, Yogesh Nimonkar, Shalaka K. Patil, Ashvini Chauhan, Amit Yadav, Dheeraj P. Dhotre, Dilip R. Ranade

**Affiliations:** ^1^National Centre for Microbial Resource (NCMR), National Centre for Cell Science (NCCS), Pune, India; ^2^Environmental Biotechnology at the School of the Environment, Florida A&M University, Tallahassee, FL, United States

**Keywords:** landfill, leachate, methanogens, anaerobes, contamination

## Abstract

The landfill is a cheap way of solid waste management in developing countries. The majority of landfills are non-sanitary and work as open garbage dumping sites and pose threats to public and environmental health. Therefore, an in-depth understanding of the chemistry and microbiology of landfills is imperative to develop the right policies for landfill management. In the current study, we investigated the chemistry and microbiology of three Indian landfill sites using culture-based and culture-independent molecular approaches. Our data indicate that the nature of landfills varies from site to site in terms of chemistry, pollutants, and pathogens. We also enriched and cultivated three methanogens using an optimized medium and constructed two high-quality draft genomes from enriched microbiomes using metagenome-assembled genome approaches. The phylogenomic study of one draft genome showed the highest 93% sequence similarity with members of *Methanomassiliicoccaceae* and was always enriched with *Acholoplasma* and *Anaerohalosphaera lusitana*. Despite all the efforts, we did not isolate it in pure culture and hypothesized that for the cultivation of some not-yet-cultured methanogen, the presence of other organisms plays an important role, and their syntrophic interaction must be discerned for its successful cultivation in the future. Co-cultivation of amino acid-degrading organisms indicates that their co-culture can assist in boosting the growth of methanogens. In addition, our data indicated that landfill leachate contains a heavy load of pollutants and treatment is a must before discharge in nature or use in irrigation or biofertilizer.

## Introduction

Due to population growth, urbanization, industrialization, and economic development, managing municipal solid waste is an emerging challenge for municipal corporations (Rawat and Ramanathan, 2011; Ajay et al., 2022; Fazzo et al., 2023). Landfilling is a cheap municipal solid waste management method in developing nations (Nimonkar et al., 2023). Landfills act as anaerobic digesters where sequential microbiological processes such as hydrolysis, acidogenesis, acetogenesis, and methanogenesis decompose the biodegradable waste and produce greenhouse gases (CH_4_, CO_2_, and N_2_O) and other toxins and pollutants. Landfills are divided into different categories based on the type of waste, including municipal waste landfills, sanitary landfills (Lambolez et al., 1994), industrial landfills, hazardous waste landfills, and demolition waste landfills (Yuan and Shen, 2011; Iravanian and Ravari, 2020). Landfill sites are the third largest anthropogenic emitter of greenhouse gases, mainly methane, which later contributes to global warming. Various factors such as the physiochemical nature of waste, the age of the landfill, the density of solid wastes at landfill sites, the depth of landfills, precipitation intensity, temperature, and microbial community structure play an important role in waste decomposition and landfill gas production (Themelis and Ulloa, 2007; Kumar and Samadder, 2017; Kumar and Agrawal, 2020).

In India, landfilling is one of the preferred sources of municipal solid waste (MSW) management (Sagar et al., 2022; Nimonkar et al., 2023). Although the percentage of sanitary landfills is increasing, to mitigate climate change and public health issues, some landfills still need to be adequately managed (Nair, 2021). Indian data about municipal solid waste composition indicates that the quantity of biodegradable waste in the total composition of MSW decreased from 2005 to 2011, and the amount of plastic and rubber increased (NITI Aayog, 2015). However, the degradation of biodegradable waste at open landfills is the main culprit for environmental health and climate change (Parvin and Tareq, 2021; Ma et al., 2023; Pan et al., 2023). Environment-friendly waste degradation strategies have always been challenging for waste management systems due to unawareness of waste disposal, inappropriate segregation, lack of advanced technologies, and irregular policy implementation. Proper segregation and processing of solid waste before discarding it at landfill sites and using biodegradable waste for biogas (biomethane) generation on the concept of waste to energy is a viable option. Generated digestate can be used as compost or fortified biofertilizer for agriculture.

Furthermore, the black-colored liquid leachate released from moisture trapped in landfills contains many pollutants and pathogens. If untreated, leachate discarded in soil or freshwater bodies causes groundwater and surface water pollution and induces eutrophication, hypoxia, fish death and the spread of pathogens in the community (Christensen et al., 1992; Reinhart and Grosh, 1998; Kjeldsen et al., 2002; Bhalla et al., 2012; Giang et al., 2018; Ferronato and Torretta, 2019; Salam and Nilza, 2021). The potential of landfill and landfill leachate in the spread of pollutants and pathogens in nearby communities and its contribution to greenhouse gas emission, ecological and soil health, contamination of surface and groundwater, effects on the release of toxic gases on garbage burning, and spread of antimicrobial resistance have been reviewed by different groups across the world including, China, Iran, South Africa, and India (Njoku et al., 2019; Nair, 2021; Parvin and Tareq, 2021; Ajay et al., 2022; Cheng et al., 2023; Fazzo et al., 2023; Ghosh et al., 2023; Kooch et al., 2023; Lee et al., 2023; Liang Y. et al., 2023; Liang Z. et al., 2023; Ma et al., 2023; Naveen, 2023; Pan et al., 2023). It has been found that microorganisms present at landfill sites and in landfill leachate play significant roles in waste degradation, greenhouse gas emission, pollutants generation, etc. Only limited data on the chemistry and microbiology of landfills are available due to the difficult nature of landfill sampling across the globe. The availability of extensive datasets on microbiology and chemistry from geographically different landfill sites will be a boon to understanding the ecological roles of landfills in climate change and public health in developing appropriate landfill management policies and improving the existing leachate and municipal solid-waste management technology.

In the current study, we have conducted a comparative analysis of physiochemical features and contamination potentials of four different landfill sites in New Delhi and Pune, India. We have performed nanoLC tandem mass spectrometry from landfill leachate and leachate sediments and reported several contaminants. In addition, we conducted enrichment for methanogenic archaea and anaerobic bacteria from the collected landfill samples and isolated different groups of obligate anaerobes and methanogens. Furthermore, we also obtained good-quality draft genomes of not-yet-cultured methanogenic archaea using a metagenome-assembled genome approach. We demonstrated that enriched methanogens, a novel genus of the family *Methanomassiliicoccaceae*, might play an important role in landfill ecology and need pure culture for better insight.

## Materials and methods

### Sample collection

Four landfill sites, located at Moshi landfill in Pune (18°39′24.8″N 73°51′28.3″E), and Gazipur (28°37′30.9″N 77°19′41.0″E) Alipur (28°44′37.3″N 77°09′32.9″E), and Okhla (28°30′43.3″N 77°16′54.0″E) landfills from National Capital Region (NCR) New Delhi, were selected for sampling purposes (Supplementary Figure 1). Sampling was done after the Monsoon in the month of November 2018. Up to 1.0 kg of degraded materials from two different depths (40–50 and 90–100 cm) of landfills were collected using soil sampling auger. Each sample was filled compactly in pre-sterile zip-locked plastic bags and sealed tightly to minimize oxygen exposure and maintain anoxic to microoxic conditions. Watery leachate and leachate sediments were collected in sterilized bottles along with the soil. All samples were transported to the laboratory on ice and immediately processed. The collected samples were homogenized separately and divided into three parts. The first part was used for the enrichment of methanogens and anaerobic bacteria. The second part was stored in a −80°C deep freezer for molecular analysis and metagenomic studies, whereas the third part was used for physiochemical analysis.

### Enrichment of methanogenic archaea

The landfills selected in this study are located in the mesophilic temperature range. Physicochemical data of sampling sites are given in Table 1. To simulate the sampling site condition, pH, salinity, and temperature of the enrichment culture were set according to site geochemistry. At the time of sampling, the temperature of the landfill was 30°C; therefore, we conducted all the enrichment and physiological experiments at 30°C to mimic the site temperature. To enrich methanogenic archaea and anaerobic bacteria, basal carbonate yeast trypticase (BCYT) medium (Touzel and Albagnac, 1983), BCYT + 30% rumen fluid, SAB medium (Khelaifia et al., 2013), and anaerobic groundwater medium were used. Basal medium was fortified with different substrates according to the targeted type of methanogens. For acetoclastic-methanogens acetate, methylotrophic methanogens methanol and hydrogenotrophic methanogens H_2_/CO_2_ were used as substrates. In brief, 30 ml medium was taken in a 50 ml capacity serum vial and made anoxic by bubbling with nitrogen gas. Vials with the pre-reduced medium were sealed using blue butyl rubber septum and aluminum crimps and sterilized in an autoclave. The serum vials headspace was filled using either filter sterilized nitrogen in case of the acetoclastic and methylotrophic type of enrichment, and hydrogen and CO_2_ gas mixture (80:20) was used for hydrogenotrophic methanogens. In total, 10% of the collected samples were inoculated in the medium and incubated at 30°C for 15 days. In the case of hydrogenotrophic methanogens, evacuation and re-pressurization of H_2_/CO_2_ in headspace are done on alternate days. Positive pressure in the headspace of each bottle indicated gas production and positive growth.

**TABLE 1:** Comparison of physicochemical data generated from different landfill samples.

S.N.	Parameters	Units	P1f	P1m	G1f	G1m	O1f	O1m	A1f	A1m
1	pH (0.5% Sol)	–	5.75	5.49	7.69	7.76	8.02	8.22	8.19	8.08
2	E.C (0.5% Sol.)	ms/cm	2.52	2.10	1.21	3.52	0.79	0.89	0.85	0.67
3	Water content	%	28.39	47.49	28.23	36.49	19.12	17.53	17.09	19.29
**(B)**	**Physical parameters**									
4	TOC	%	27.22	27.55	4.59	8.39	2.96	2.97	1.86	2.7
5	TOM	%	64.83	65.6	13.61	24.91	8.51	8.76	3.97	5.85
6	C:N ratio	–	18:39:01	19:04:01	6.81:1	8.5:1	12.59:1	12.96:1	12.06:1	10.8:1
**(C)**	**Texture**									
7	Sand	%	70.67	63.96	73.39	87.5	79.56	78.63	78.32	74.31
8	Clay	%	15.58	16.37	3.26	0.241	1.8	1.8	2.42	2.05
9	Silt	%	13.75	19.67	23.35	12.35	18.64	19.57	19.26	23.64
**(D)**	**Fertilizer value**									
10	Total Nitrogen	%	2.20	2.17	0.67	0.99	0.24	0.23	0.15	0.25
11	TON	%	1.48	1.42	0.54	0.79	0.19	0.18	0.11	0.19
12	TInN	%	0.72	0.65	0.07	0.13	0.01	0.01	0.02	0.01
**(E)**	**Heavy metals**									
13	Nickel as Ni	ppm	25.6	24	49.29	328.3	29.97	36.82	26.75	27.89
14	Lead as Pb	ppm	60.9	85.1	224.29	27.03	65.41	89.16	20.16	25.68
15	Chromium as Cr	ppm	35.98	40.7	76.66	827.5	76.54	97.86	28.8	30.31
16	Arsenic as	ppm	<0.1	<0.1	<1.0	<1.0	<1.0	<1.0	<0.1	<1.0
17	Mercury as Hg	ppm	0.1	0.145	3.24	1.04	1.35	2.21	1.96	1.81
**(F)**	**Other**		**NaCl**							
18	Sodium as Na	%	1.75	0.91	0.15	0.25	0.13	0.13	0.09	0.09
19	Chloride as Cl	%			0.06	0.01	0.04	0.04	0.04	0.02

TOC, total organic carbon; TOM, total organic matter; TON, total organic nitrogen; TInN, total inorganic nitrogen; 1M, 1metre depth; 1F, 1feet depth; P, Pune; G, Gazipur; O, Okhla; A, A lipur.

After every 2 weeks, 5 ml of culture was anaerobically transferred into a fresh medium supplemented with vancomycin to suppress bacterial growth (Hilpert et al., 1981). Positive enrichment or growth of methanogens in serum vials was confirmed by detection of methane formation in headspace using gas chromatography (GC), increase in optical density of the culture medium, flame test (burning) of headspace gas, and detection of cell fluorescence at 420 nm using fluorescence microscopy as discussed by Nimonkar et al. (2023).

In addition to methanogenic archaea, we also isolated obligate anaerobic bacteria from collected landfill samples using pre-reduced peptone yeast extract glucose (PYG), thioglycolate medium, and minimal medium with different electron donors. The enriched cultures were spread and streaked on respective medium plates in the anaerobic glove box (Thermo Fisher) at 30°C for isolation. Isolated pure cultures were preserved, according to Prakash et al. (2013). In addition to spreading on solid medium agar plates, we also tried to isolate the pure culture of obligate anaerobes and methanogens using Roll-Tube methods, as suggested by Prakash and Ranade (2020). The purity of cultures was checked by re-streaking on plates, and the obligate anaerobic nature of the strains was verified by incubating a set of similar streaked plates in aerobic and anaerobic conditions at 30°C. Inoculated plates were incubated in airtight jars filled with H_2_/CO_2_ for hydrogenotrophic archaeal isolation. Purified strains were preserved in a deep freezer, according to Prakash et al. (2013). In the case of non-isolated pure culture, the enriched consortium was preserved in the SAB medium containing glycerol at −80°C to check the potential for methane production of cultures after 45 days.

### Physiochemical analysis

All collected samples from both depths of the different landfill sites were subjected to physiochemical analysis, as discussed by Nimonkar et al. (2022). Different physiochemical parameters of the samples such as total organic carbon, organic matter, C: N ratio, heavy-metal contents, total organic nitrogen, total inorganic nitrogen, texture, and volatile fatty acids (VFA) were analyzed using standard practice as mentioned by Nimonkar et al. (2022). In addition, salinity, pH, electrical conductivity, water content, and texture of samples were also checked. Landfill leachate and leachate sediment samples were extracted and analyzed for pollutants and contaminants using nano-LC tandem mass spectrometry (n-LC-MS/MS), as discussed by Sanders and Edwards (2020). In brief, metabolites from landfill leachate and leachate sediments were extracted using water:acetonitrile (1:1) as extraction solvent, followed by centrifugation for 10 min at 10000 rpm at 4°C to settle the particulate and other coarse materials. After that, the supernatant was dried in Thermo SC250EXP SpeedVac Concentrator (Thermo Fisher Scientific-CA) at 30°C and used for liquid chromatography-tandem mass spectrometry analysis (nLC-MS/MS). Before analysis, dried samples were rehydrated in 500 μl of 0.1% formic acid in MS-grade water. Rehydrated samples were passed through a 0.45 μm filter taken in brand new, unused glass vials and subjected to nLC-MS/MS analysis. An externally calibrated Thermo Q-Exactive HF (high-resolution electrospray tandem mass spectrometer) was used with the Dionex UltiMate3000 RSLCnano System. Operation conditions of nano-LC tandem mass spectrometry (n-LC-MS/MS) were discussed by Sanders and Edwards (2020).

### Whole genome sequencing and analysis

We observed that some enrichment for hydrogenotrophic methanogens showed positive growth of organisms and produced methane in the headspace as detected by gas chromatography. However, we never successfully isolated the organism in pure culture. We used a shotgun metagenome sequencing and metagenome-assembled genomics (MAGs) approach to reconstruct the genome from such an enriched microbiome. We conducted metagenome sequencing using the Illumina Nextera Flex kit on the MiSeq 2000 platform. The raw read quality assessment was done using the FastQC v0.11.5 tool. The assessed raw reads were used for the assembly construction using metaSPAades v3.11.1 (Nurk et al., 2017). Then, the raw reads were mapped on an assembled metagenome to get the scaffold coverage needed for the genome reconstruction.

The metagenome assembly and alignment files generated in the previous step were used by MetaBAT v2.12.1 to generate the bins (Kang et al., 2015). In the subsequent steps, these bins were considered reconstructed metagenome-assembled genomes (MAGs). The reconstructed genomic bins were validated using CheckM v1.0.11 (Parks et al., 2015). The 16S rRNA was predicted using a standalone version of rammer-1.2. The obtained 16S rRNA gene sequences were searched in the EzBioCloud 16S database (Yoon et al., 2017). The gene prediction for the individual bin was done using polka 1.12, and obtained coding sequences were subjected to the UniProt/SwissProt database using diamond v0.9.22.123 blast utility. The matched protein IDs were subjected to the UniProt ID mapping tool to discover the pathways and gene ontology associated with the protein sequence. The DNA sequence quality of both genomes was checked using FastQC and the raw sequences were assembled using the *de novo* assembler MIRA 4.0 (Chevreux, 2007). Assembled draft genomes of both organisms were annotated by the RAST annotation server and PATRIC Genome Annotation Pipeline (Overbeek et al., 2014; Wattam et al., 2014). The average nucleotide identity (ANI) implemented in OrthoANI was computed on homologous genes for all pairs of genomes (Lee et al., 2016). The genomes were reconstructed from the metagenome (bin 4 and bin 26, respectively) of landfill leachate to find the genes likely involved in H_2_ and methane production. Reconstructed genome characteristics are shown in Table 2.

**TABLE 2:** Genomic statistics from draft genome sequence constructed using a metagenome-assembled genomics approach.

	Bin 4	Bin 26
Identification	*Anaerohalosphaera* *lusitana*	*Methanomassiliicoccus* *luminyensis*
Contigs	719	44
CDS	1939	1408
tRNA	16	26
rRNA	1	2
Genome length (bp)	26004132	1985171
GC%	55	62
N50	3942	53894
Hypothetical proteins	1259	911
CRISPR	0	1
Proteins GO assignments	303	210

### Molecular phylogeny and average nucleotide identity (ANI) analysis

**FIGURE 1:**
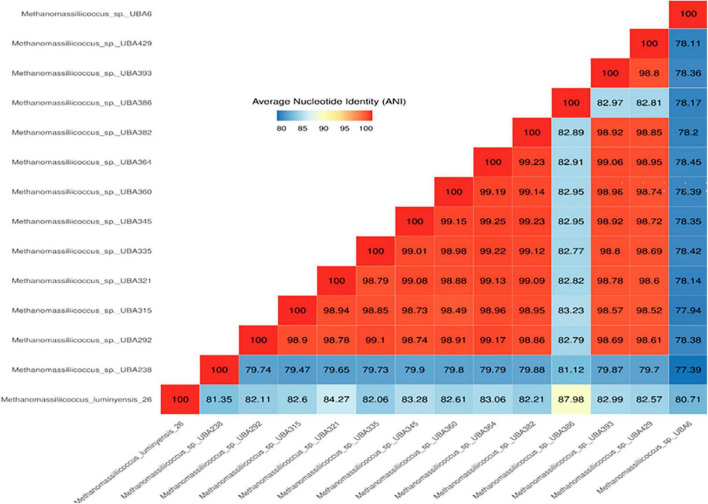
Representation of ANI values of different strains of methanogens show close similarity with the genome obtained from this study.

**FIGURE 2:**
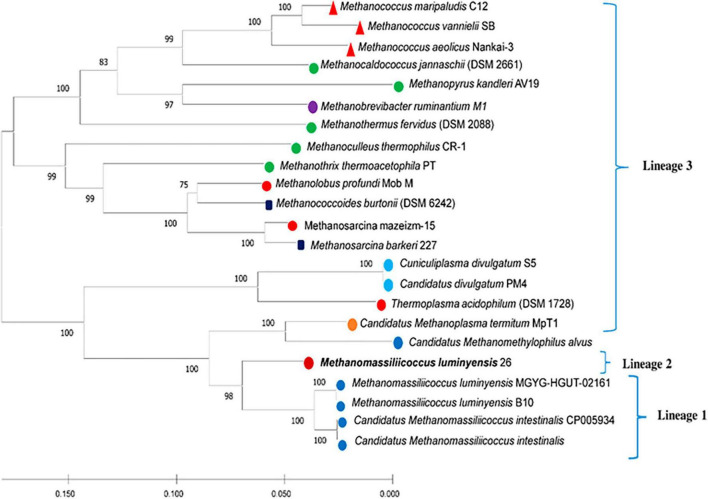
Neighbor-joining phylogenetic tree construction based on 16S rRNA gene sequence. Selected sequences are labeled according to their habitats (landfill leachate sludge, mesophilic, thermophilic, diverse environment, freshwater, extremely acidophilic, termite gut, rumen gut, and human gut). Bootstrap support values are shown as a percentage before the respective nodes; the scale bar indicates the number of substitutions per site. Phylogenetic clusters are indicated on the right of the tree. *Methanomassiliicoccus luminyensis* 26 of tree is proposed “*Candidatus Methanomassiliicoccus indica*.”

**FIGURE 3:**
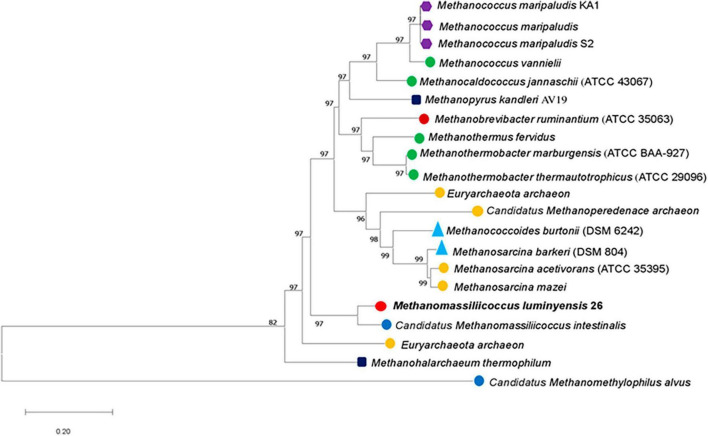
Evolutionary relationships of methyl-coenzyme M reductase (McrA) of different methanogens. The evolutionary history was constructed using the neighbor-joining method. The analysis involved 21 amino acid sequences of mcrA genes. MEGA7 Kumar et al. (2016). Bootstrap support values are shown as a percentage before the respective nodes; the scale bar indicates the number of substitutions per site. Phylogenetic clusters are indicated on the right of the tree. *Methanomassiliicoccus luminyensis* 26 of tree is proposed “*Candidatus Methanomassiliicoccus indica*.”

Phylogenetic positions of enriched methanogen were studied using 16S rRNA, *mcr*A, and whole genome sequence-based phylogeny, as well as by calculating the average nucleotide identity (ANI) using a constructed draft genome (Figures 1–3). For ANI analysis, all *Methanomassiliicoccus* spp. genome sequences were downloaded from the NCBI genome browser. In brief, as mentioned earlier, the draft genome sequence of methanogens was obtained from enriched culture using metagenome-assembled genomics. Nearly full-length 16S rRNA and *mcr*A gene sequences were extracted from a constructed high-quality draft genome. We obtained a comprehensive set of 16S rRNA and *mcr*A gene sequences showing close relatedness with obtained sequences from draft genomes using public databases comprising all significant lineages of methanogens. The evolutionary history was constructed using the neighbor-joining method in MEGA7 (Kumar et al., 2016). All the phylogenomic and gene-based phylogenetic studies were conducted, as recommended by Prakash et al. (2023).

#### Data and sequences

BioSample accession for *Anaerohalosphaera lusitana* is SAMN38030365 while BioSample accession for *Methanomassiliicoccus luminyensis*-26 is SAMN38029128. In addition, the GenBank accession numbers for the 16S rRNA gene sequence of cultivated strains are as follows: OR342400, OR342399, OR342402, OR342395, OR342396, OR342401, OR342406, OR342403, OR342397, OR342394, OR342405, OR342406, OR342398, OR835591, OR835592, OR835593, and OR835594.

## Results

### Physicochemical features and isolates

Physicochemical data of selected landfill sites from different depths are given in Table 1. The pH of the sites varies from 5.4 to 8.2, and the electrical conductivity (EC) ranges from 0.6 to 2.1. In contrast, the’ water content of the sample was higher in the lower depth than in the upper depth. Similarly, we also observed depth and site-wise variations in total organic carbon (TOC), total organic matter (TOM), and carbon and nitrogen ratio. Texture-wise, all the samples from all the depths showed more sandy nature followed by silt and clay. The total nitrogen content of Pune’s site was higher than that of others. Nickel, lead, and chromium contents were higher than other heavy metals at all the sites. In addition to solid landfill samples, analysis of watery leachate and sediment of landfill leachate indicates a considerable load of pollutants in both samples. Approximately 4000–4500 different peaks of compounds have been detected from leachate and leachate sediments. In contrast, 500 to >1500 different compounds and pollutants were detected in watery Leachate (Supplementary Figure 2). They constitute different classes of xenobiotics belonging to mono- and polycyclic-aromatics hydrocarbons (PAHs), polychlorinated biphenyls (PCBs), polybrominated biphenyls, pesticides (herbicides, fungicides, and insecticides), residual pharmaceuticals (beta-blocker, analgesics, antibiotics, antiseptics, and estrogenic drugs), heavy metals (mercury, nickel, arsenic, zinc, and cadmium), plasticizers, endocrine disruptors, and hormones. Nitrate, nitrite, ammonia, and phosphate were also detected in high quantities, and their levels were higher than the maximum permissible limit.

We confirmed the positive growth of methanogens in enrichments by confirmation of methane accumulation in the headspace of enrichment vials using gas chromatography (GC), increased cell turbidity of culture medium, and detection of fluorescence in enriched cells under the microscope due to the presence of cofactor F_420_ in methanogens (Nimonkar et al., 2023). Although we successfully enriched the methanogens from all enrichments (Supplementary Figure 3) except *Methanosarcina*, we could not cultivate other methanogens in pure culture. During the enrichment, we added vancomycin to suppress the growth of bacteria and provided a condition that is conducive only to methanogens. However, we also detected some anaerobic bacteria in our enrichments and were able to cultivate them on solid medium agar plates. A list of the cultivated organisms from different enrichment is given in Table 3. We have isolated and purified *Clostridium thiosulfatrieducens*, *Clostridium saccharolyticum*, *Aminobacterium colombiense*, *Proteiniborus indolifex*, *Acholeplasma palmae*, *Cloacibacillus porcorum*, *Vagococcus acidifermentans*, *Cutibacterium avidum*, *Aminobacterium colombiense*, *Sphaerochaet aassociata*, *Bacillus luti*, *Clostridium sporogenes*, *Clostridium beijerinckii*, *Paraclostridium benzoelyticum*, *Clostridium diolis*, *Clostridium beijerinckii*, *Clostridium diolis*, and *Methanosarcina mazei* from different enrichment of current study. In addition to isolating the above organisms, we have cultivated and isolated the members of *Acholeplasma* from methanogenic enrichment vials. However, we observed that after two or three generations of cultivation, the strain could not survive, and we could not maintain it as an active culture. Similarly, except *for Methanosarcina mazei*, we could not isolate the other methanogens present in the enrichment and studied them using the approach of metagenome-assembled genomes.

**TABLE 3:** List of isolated pure cultures of anaerobic bacteria and archaea obtained from different enrichment.

S.N.	Strain ID/accession no.	Sites	Closest match	Sequence size (bp)	Isolation site	References
1	YSNML-9 OR342400	Pune (1F)	*Clostridium subterminale* DSM 6970 ^(T)^ AF241844 (99.8%)	1,383	Multiple strains from various sites	Sedlar et al., 2021
2	YSNML-8 OR342399	Pune (IF)	*Clostridium thiosulfatireducens* LUP 21 ^(T)^ AY024332 (99.8%)	1,225	Anaerobic sludge blanket reactor	Hernández-Eugenio et al., 2002
3	YSNML-14 OR342402	Pune (1M)	*Lacrimispora saccharolytica* WM1 ^(T)^ CP002109 (99.9%)	1,350	Sewage sludge	Parte et al., 2020
4	YSNML-2 OR342395	Pune (1M)	*Aminobacterium colombiense* DSM 12261 ^(T)^ CP001997 (99.8%)	1,379	Anaerobic sludge	Baena et al., 1998
5	YSNML-7 OR342398*	Pune (1F)	*Proteiniborus indolifex* BA2-13 ^(T)^ KT351641 (96.1%)	1,330	Thermophilic industrial-scale biogas plant	Hahnke et al., 2018
6	YSNML-4 OR342396*	Pune (1M)	*Mariniplasma anaerobium* MaBG01hy22 ^(T)^ LC430819 (92.8%)	1,437	Sulfidic bottom water of a shallow brackish meromictic lake	Watanabe et al., 2021
7	YSNML-11 OR342401*	Pune (1F)	*Cloacibacillus porcorum* CL-84 ^(T)^ CP016757 (89.9%)	1,144	Swine intestinal tract	Looft et al., 2013
8	YSNML-21 OR342406	Pune (1F)	*Vagococcus acidifermentans* AC-1 ^(T)^ FJ211190 (99.7%)	1,324	Bioreactor treating food wastewater	Wang et al., 2011
9	YSNML-15 OR342403*	Pune (1F)	*Cutibacterium avidum* ATCC 25577 ^(T)^ AGBA01000019 (99.6%)	1,302	Skin	NA
10	YSNML-5 OR342397	Pune (1M)	*Aminobacterium colombiense* DSM 12261 ^(T)^ CP001997 (99.6%)	1,363	Anaerobic sludge	Baena et al., 1998
11	YSNML-1 OR342394*	Pune (1F)	*Sphaerochaeta associata* GLS2 ^(T)^ JN944166	589	Cultures of Methanosarcina mazei	Troshina et al., 2015
12	YSNML-16 OR342405	Pune (1F)	*Clostridium sporogenes* DSM 795 ^(T)^ JFBQ01000001 (98.9%)	1,434	Human feces	Poehlein et al., 2015
13	YSNML-20 OR342406	Pune (1M)	*Vagococcus acidifermentans* AC-1 ^(T)^ FJ211190 (100%)	1,442	Acidogenic fermentation bioreactor	Wang et al., 2011
14	AnPYGLec01 OR835591	New Delhi (1F)	*Clostridium beijerinckii* DSM 791 ^(T)^ X68179 (99.9%)	1,091	Multiple strains from various sites	Keis et al., 2001
15	AnPYGL1mt2b OR835592	New Delhi (1F)	*Paraclostridium benzoelyticum* JC272 ^(T)^ LBBT01000182 (100%)	642	Marine sediment	Sasi Jyothsna et al., 2016
16	AnPYGLec07 OR835593	New Delhi (1F)	*Clostridium beijerinckii* DSM 791 ^(T)^ X68179 (99.5%)	1,277	Multiple strains from various sites	Keis et al., 2001
17	ArcL1mtmeth11 OR835594	New Delhi (1M)	*Methanosarcina mazei* S-6 ^(T)^ CP009512 (100%)	672	Sewage sludge plant	Mah and Kuhn, 1984

Data in parentheses showed% sequence similarity. 1M, 1metre depth; 1F, 1feet depth of sampling; NA, not available.

*Data taken from Nimonkar et al. (2022).

### Molecular phylogeny and average nucleotide identity (ANI) analysis

**FIGURE 4:**
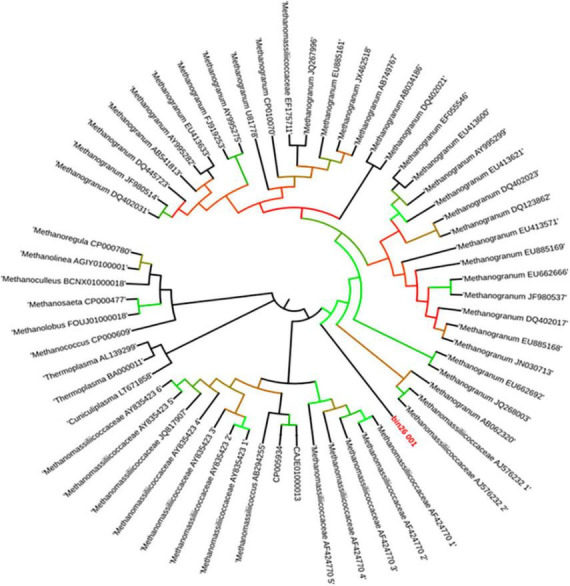
Phylogenetic tree constructed using phylogenomic approach from draft genome sequence obtained from this study and its close relatives.

Average nucleotide identity (ANI) has been an accepted criterion for prokaryotic species delineation, and it has replaced wet-lab-based DNA–DNA hybridization (Goris et al., 2007). According to current standards, newly discovered strains are considered to belong to the same species if their 16S rRNA gene sequence identity is >98.5% and their ANI is >95% (Goris et al., 2007). Thus, the genome constructed from enrichment under this study shows less than <95% ANI identity with human fecal-associated *Methanomassiliicoccus luminyensis* B10 (Figure 4). It indicates that the strain of *Methanomassiliicoccus* enriched from Indian landfills is different and a novel species of the genus *Methanomassiliicoccus* (Figure 1).

Similar to the ANI result, the 16S rRNA gene sequence similarity and phylogenetic analysis data indicate that the draft genome sequence of Bin-26 showed the highest sequence similarity with not-yet-cultured *Methanomassiliicoccus* species and phylogenetically different. 16S rRNA-based phylogenetic tree showed the relationship with human-associated members of *Methanomassiliicoccus* isolated from stool samples and distantly related with methanogens isolated from fresh water and extreme environment (Figure 2). Furthermore, phylogenetic tree constructed using *mcr*A genes obtained from methanogens cultivated from different habitats including the animal gut, thermophile, mesophile, hypersaline freshwater, and human-gut isolated *Candidatus Methanomassiliicoccus intestinalis* (Figure 3) also substantiate the findings of 16S rRNA gene-based phylogeny and sequence similarity. It showed that enriched methanogens tightly coupled with *Candidatus Methanomassiliicoccus intestinalis.* The phylogenetic tree constructed using whole genome sequences indicates that this organism is different and supports the finding obtained from sequence similarity search, ANI analysis, 16S rRNA, and *mcr*A gene-based phylogenies.

Thus, based on our sequence similarity search, ANI calculation, and phylogenetic analysis, we found that enriched methanogens are cultured for the first time, but not-yet-isolated novel methanogens from landfill leachate and sludge. It is phylogenetically similar to methanogens isolated or characterized from human origin. It also indicates the relationship of fecal contamination of landfills, which is quite common because community-disposed diapers and pads reach municipal landfill sites along with other solid waste.

### Proof of putative methane formation pathways in “*Candidatus Methanomassiliicoccus indica*”

**FIGURE 5:**
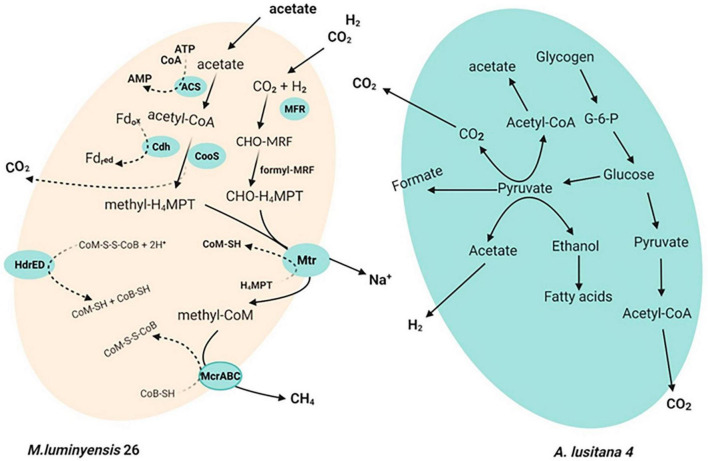
A putative illustration of syntrophic mechanism: Methanogens employ two separate pathways to convert CO_2_ into methane: either the methyl group of acetate or the reduction of carbon dioxide with electrons from hydrogen or format. Hence, CO_2_ and acetate are generated in the fermentation of glucose and glycogen. Biochemical pathway of H_2_-dependent reduction of CO_2_ to CH_4_. The C1 moiety is transferred from CO_2_ via MF, H_4_MPT, and CoM (CoM-SH) into CH_4_. Details of putative proteins of acetotrophic pathways in *M. luminyensis* 26. Electron-donating species formate is produced by the anaerobic degradation of organic matter by fermenting and syntrophic bacteria. Enzyme abbreviations are as follows: Fd, ferredoxin; HydABC, bifurcating [Fe-Fe] hydrogenase; HyaABC, [NiFe] hydrogenase; Acs, acetyl-coenzyme A synthetase; CooS, carbon monoxide dehydrogenase; CdhA, acetyl-CoA decarbonylase/synthase complex; Mtr, methyl-tetrahydromethanopterin: CoM methyltransferase; McrABG, methyl-coenzyme M reductase; HdrED, coenzyme B-coenzyme M heterodisulfide reductase; FpoABDHIJKLMNO, F_420_H_2_ dehydrogenase.

An earlier report suggests that the closest relative of our cultured methanogens, *M. luminyensis*, uses a hydrogenotrophic pathway for methane production. Annotation of constructed genomes demonstrated that our enriched methanogens from this study use hydrogenotrophic metabolism for methanogenesis (Figure 5). The putative pathway of hydrogenotrophic methanogenesis in our enriched methanogens is demonstrated in Figure 5. Furthermore, the annotation result of another constructed genome showing sequence similarity with *Anaerohalosphaera lusitana* 04 indicates that this organism has the potential to generate CO_2_ and H_2_ (Figure 5). Thus, the co-cultivation and enrichment of *Anaerohalosphaera lusitana* 04 and “*Candidatus Methanomassiliicoccus indica”* from our enrichment indicate that these two organisms might use a syntrophic lifestyle and use H_2_ and CO_2_ generated by *Anaerohalosphaera lusitana* 04 during enrichment (Figure 5 and Supplementary Figure 3).

We also propose that *“Candidatus Methanomassiliicoccus indica”* might have the ability to utilize available acetate for acetoclastic way methanogenesis because it harbors acetyl-CoA synthetase for acetate activation, CO dehydrogenase/acetyl-CoA synthase (CODH/ACS) to oxidatively split acetyl-CoA into CO_2_ and CH_3_-H_4_MPT, tetrahydromethanopterin *S*-methyltransferase, and methyl-CoM reductase for methyl-CoM reduction to CH_4_. To couple acetyl-CoA oxidation and reductive CH_4_ generation, “*Candidatus Methanomassiliicoccus indica”* could transfer electrons from reduced ferredoxin to coenzyme M (CoM-SH) and coenzyme B (CoB-SH). We also identified an FpoF-lacking F_420_H_2_ dehydrogenase (Fpo) complex and heterodisulfide reductase (HdrDE) that are known to generate an ion motive force. Thus, based on the above evidence, we hypothesized that “*Candidatus Methanomassiliicoccus indica”* can also use acetoclastic methanogenesis for energy conservation. The genomic features of the constructed genome of *Anaerohalosphaera lusitana* 04 and “*Candidatus Methanomassiliicoccus indica*” are given in Table 2 and Supplementary Figures 4, 5, respectively.

## Discussion

Landfills are one of the main contributors to global methane emissions and are responsible for the problem of global warming and climate change. They contribute to greenhouse gas emissions, and unmanaged landfill plays a significant role in the spread of pollutants and pathogens to the natural ecosystem and groundwater by the process of discharge and recharge, respectively, and threats to the concept of sustainability. Knowledge of landfill microbiology and chemistry is imperative to mitigate methane at a source point and properly manage landfills from environmental and energy perspectives. Our physicochemical data indicate that landfills have immense surface and groundwater contamination potential. Landfills and their generated leachate contain a huge load of pollutants and pathogens, and we should treat them appropriately before discarding them in the natural environment (Nimonkar et al., 2022; Sagar et al., 2022). We should nourish the concept of a sanitary landfill, which promotes the collection of generated methane and leachate that help mitigate climate change and environmental toxicity.

We have compared the physiochemical data of landfill leachate obtained from this study with other studies conducted on landfill leachate, including Canada (Kochany and Lipczynska-Kochany, 2009); Bandung, Indonesia (Septiariva et al., 2019); and Gazipur, New Delhi (Kumari et al., 2019) and Mavallipura, Bengaluru, India (Naveen et al., 2018). Although the extensive characterization of landfill leachate in other studies has yet to be conducted and is limited to only a few basic features, comparative analysis of available data on similar physiochemical traits indicates substantial variations. It might be due to different climates, geographical locations, age, nature of waste dumping practices, etc. Therefore, an extensive analysis of physicochemical features is imperative to improve the existing leachate treatment technology or to develop a new one.

High total organics, high electrical conductivity, and low pH of Pune landfills from both the depths than all the selected landfill sites of New Delhi indicate that the Pune landfill site contains more wet organic wastes, which produced higher concentrations of organic acids after microbial degradation, lowering the pH. While high pH, low TOC, and EC of New Delhi sites indicate less organic waste is going on dumping sites, practices of separating the dry and wet waste are better than those in Pune. Another possible reason could be the higher rate and longer duration of precipitation in Pune, which provides the ideal condition for microbial degradation of waste compared to the dry weather of New Delhi.

Furthermore, the higher concentration of arsenic and lead in the New Delhi landfill than in Pune might be due to differences in geographical location, the physicochemical nature of garbage and waste, and high levels of contamination of industrial waste. Data obtained from landfill leachate and leachate sediments by extraction and nLC-MS/MS analysis indicate more elevated contaminant levels in sediments than in watery leachate. High contaminants in sediment are an obvious observation because non-soluble pollutants and chemicals gradually accumulate in sediment by sedimentation processes, while watery leachate retains only soluble chemical constituents. Thus, our data indicate that landfill leachate sediments and watery leachate are highly contaminated and should only be used for agricultural irrigation or environmental discharge with adequate treatment.

We were surprised to get vast amounts of contaminants during our MS/MS leachate analysis. Due to the high resolving capacity of nano-LC compared to standard LC and the high discriminatory power of MS/MS, we got more metabolites in the leachate sample. We tried to present comparative leachate data from other landfill sites across the globe; however, we got information only from Canada, Indonesia, and two landfills in India, one of which was from our group (Kochany and Lipczynska-Kochany, 2009; Naveen et al., 2018; Kumari et al., 2019; Septiariva et al., 2019). The data available from other studies are limited to pH, TOC, BOD, and COD, and few more parameters and extensive data for comparison are lacking. Detection of the high levels of pollutants and heavy metals from this study supported previous data generated from landfill and landfill leachate in different studies conducted in the past (Baldacci et al., 2018; Parvin and Tareq, 2021; Siddiqua et al., 2022; Nimonkar et al., 2023). Detection of various classes of contaminants from landfill and landfill leachate indicate that different kinds of mixed wastes discarded and dumped in municipal solid wastes, including industrial, hospital, pharmaceuticals, household, personal care products, electronics, batteries, ceramics, glass, paint, and agrochemicals. These wastes contain residual levels of leftover amounts of these contaminants that reach and accumulate in leachate and contaminate the natural ecosystem. However, we presented a very brief overview of the classes of pollutants detected in leachate in the present manuscript. This manuscript gives a quick overview of leachate contamination level, its ecosystem toxicity, and the need for adequate treatment before discarding the natural ecosystem from a public and environmental health perspective.

In this study, we aimed to cultivate and study novel methanogens from different landfill sites. In addition to methanogens, we have cultivated several groups of obligate anaerobic bacteria, which grew along with methanogenic enrichment. It indicates that the conditions used for methanogenic enrichment promoted the growth of these kinds of microbes. A list of cultivated obligate anaerobes dominated by the members of the genus *Clostridium* is given in Table 3. Several species of *Clostridium* were obtained from methanogenic enrichment, which indicates that the cultivation condition of methanogens also favors the growth of *Clostridium* (Nimonkar et al., 2022). It also indicates that *Clostridium* plays an essential role in the ecology of landfills and actively participates in waste degradation in the lower anoxic part of landfills and provides substrates such as volatile fatty acids and hydrogen for methanogenesis (Lee et al., 2014). Similar to our observation, past studies on landfills also supported the role of the members of the genus *Clostridium i*n landfill waste degradation. We have isolated *Aminobacterium columbine* from this study, which has the potential to degrade amino acids anaerobically. The past data indicated that hydrogenotrophic methanogens are an essential partner of *Aminobacterium*, and co-cultivation with the hydrogenotrophic methanogen boosts its substrate utilizing potential (Baena et al., 1998, 2000; Hamdi et al., 2015). Another anaerobic bacterium *Proteiniborus indolifex is also*, isolated in this study. Similar to *Aminobacterium columbine*, it degraded proteins and amino acids and was reported from methanogenic co-culture (Hahnke et al., 2018). *Cloacibacillus porcorum* also showed similar physiology and metabolism to the above two (Looft et al., 2013). *Vagococcus acidifermentans* was previously isolated from habitats prone to waste degradation (Wang et al., 2011), while *Cutibacterium avidum* is an emerging pathogen. In addition, *Sphaerochaet* associate was previously isolated from cultures of *Methanosarcina mazei* and indicated both are closely associated. Thus, analysis of metabolism, site of isolation, and nature of substrate utilization indicate that these organisms are amino acids or protein fermenters associated with methanogens and methanogenic activities. Although the syntrophic relationship of these anaerobic bacteria and methanogenic archaea is understudied, it is a good subject matter for future in-depth study, which can boost the cultivation of methanogens on the concept of co-culture of these organisms.

Furthermore, despite all the efforts, we were unable to cultivate the methanogens showing a sequence similar to *Methanomassiliicoccus luminyensis*, and we noticed that enrichment was always associated with *Anaerobalosphaera and Acholeplasma* showing similarity with *Acholeplasma palmae.* We cultured this *Acholeplasma* but lost its viability after 2–3 sub-culturing, and we did not maintain the pure culture. In addition, we also constructed a draft genome sequence of *Anaerobalosphaera lusitana* from this study. From draft genome data, we hypothesized that the presence and syntrophic interaction of *Anaerobalosphaera lusitana* and *Acholeplasma* are essential for the growth of organisms enriched during this study.

Data obtained from the genome sequence are shown in the genomic statistics Table 2. Phylogenetic analysis using a whole genome sequence comparison (phylogenomics) approach, 16S rRNA gene sequences, average nucleotide identity (ANI), and methyl-coenzyme M reductase (*mcrA*) based approach indicated that the enriched methanogens are members of the family *Methanomassiliicoccaceae*, with type genus of the family is *Methanomassiliicoccus*. *Methanomassiliicoccus luminyensis* is the only cultivated representative available. Metagenomic data indicated that members of *Methanomassiliicoccus* were detected abundantly at landfill sites and preferably used methylotrophic type of metabolism. Constructed draft genome using metagenome-assembled genomics from this study showed only 93% sequence similarity and 81.3% ANI-value based on whole genome sequence comparison with its closest relative. 16S rRNA and *mcrA*-based phylogeny also substantiate the same finding.

The phylogenetic tree constructed using *mcrA* genes showed its closeness with *Candidatus Methanomassiliicoccus intestinalis.* In contrast, 16S rRNA-based phylogeny showed similarity with *Candidatus Methanomassiliicoccus intestinalis* and *Methanomassiliicoccus luminyensis* but did not cluster with any of them. Thus, whole phylogenomic data indicate that the organism enriched by us needs to be isolated in pure culture and distantly related to previously studied pure culture and sequences of the members of the family *Methanomassiliicoccaceae*. Based on methane formation in the enrichment, substantial differences in phylogeny, average nucleotide identity, and sequences of *mcrA* and 16S rRNA genes, we proposed it as “*Candidatus Methanomassiliicoccus indica*” enriched from Indian landfill sites. We observed that the enrichment of this organism was always associated with *Anaerohalosphaera lusitana* and other obligate anaerobic bacteria. Although we did not isolate this organism even after several attempts and hypothesized that the co-culture of other organisms might help in the successful cultivation of this organism. We also hypothesized that isolation and cultivation in pure culture need of syntrophic relationships or metabolites by others are imperative. Cultivated methanogens might play an important role in landfill ecology and methane generation and can serve as ideal seed cultures for biogas generation using bio-methanation processes. Further study on its in-depth characterization and understanding at physiological levels is imperative.

It is reported that anaerobic degradation of organic matter by fermenters and syntrophic bacteria produces hydrogen (H_2_). Syntrophic bacteria further degrade the alcohols and fatty acids to acetate, H_2_, and CO_2_ (Conrad et al., 1989; Schink, 1997). Acetate and H_2_ or formate plus CO_2_ eventually serve as substrates for methanogens. This study found polysaccharide (especially glycogen) fermentation genes in *the Anaerobalosphaera lusitana* 04 genomes. It seems that *A. lusitana* 04 could contribute to polysaccharide fermentation, resulting in alcohols, fatty acids, and H_2_ production (syntrophic bacteria). The bacteria further degrade the alcohols and fatty acids to acetate, H_2_ (alternatively formate), and CO_2_. Thus, syntrophically, this acetate and H_2_ plus CO_2_ finally serve as substrates to “*Candidatus Methanomassiliicoccus indica*” for methane production via autotrophic (acetoclastic) or hydrogenotrophic, respectively, shown in Figure 5. A pure culture study of *Anaerohalosphaera lusitana* has supported the above hypothesis generated by genome sequence data. It is found that *Anaerohalosphaera lusitana* produced ethanol, acetate, and H_2_ as the main end product by anaerobic fermentation of d-glucose (Pradel et al., 2020), which can support the growth of *Methanomassiliicoccus*, which is used hydrogen as an electron donor for the reduction of other substances.

## Data availability statement

The datasets presented in this study can be found in online repositories. The names of the repository/repositories and accession number(s) can be found in the article/Supplementary material.

## Author contributions

OP: Writing – original draft, Conceptualization, Investigation, Project administration, Supervision, Writing – review and editing. SP: Methodology, Data curation, Writing – review and editing. YN: Methodology, Data curation, Writing – review and editing. SD: Methodology, Data curation, Writing – review and editing. AY: Writing – review and editing. DD: Writing – review and editing. DR: Writing – review and editing. AC: Data curation, Writing – review and editing.
