# Supplementary figures and images for: Culture-based and culture-independent approach for the study of the methanogens and obligate anaerobes from different landfill sites
Source: Front Microbiol. 2024 Jan 29;14:1273037. doi: 10.3389/fmicb.2023.1273037 (PMC10860756; doi:10.3389/fmicb.2023.1273037)

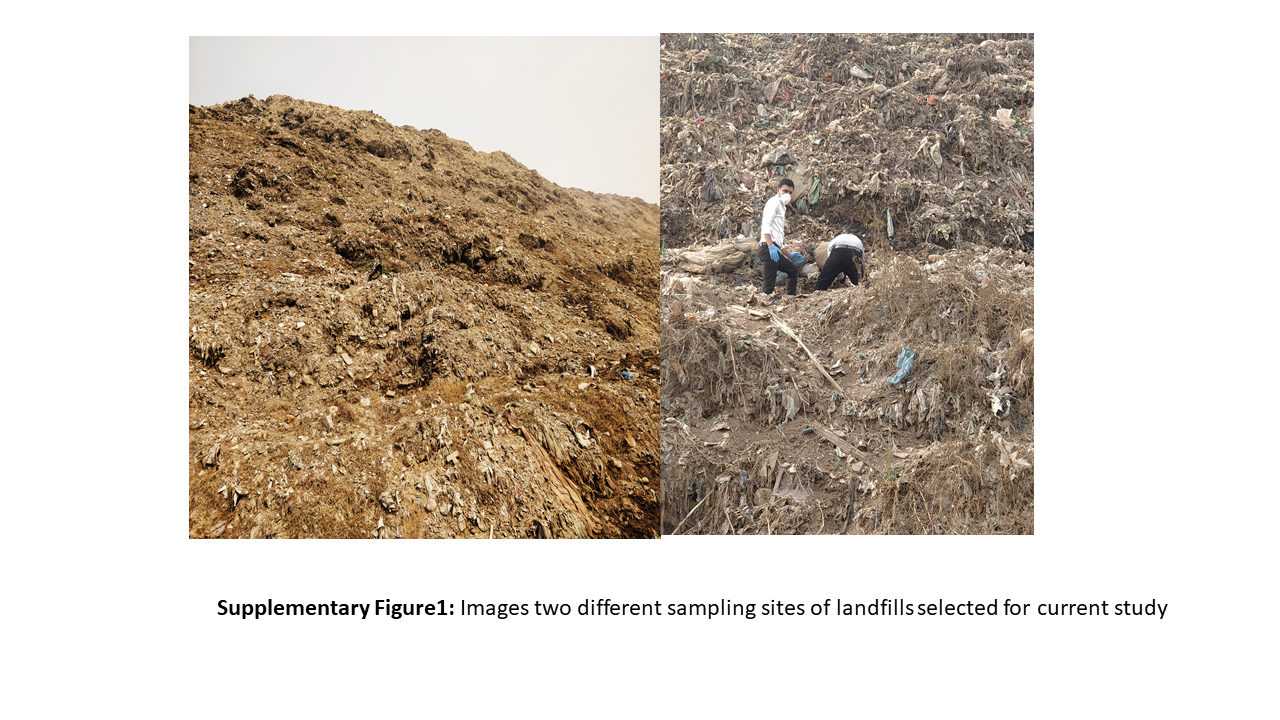

Supplement: Supplementary file 1 [file Image_1.tif]

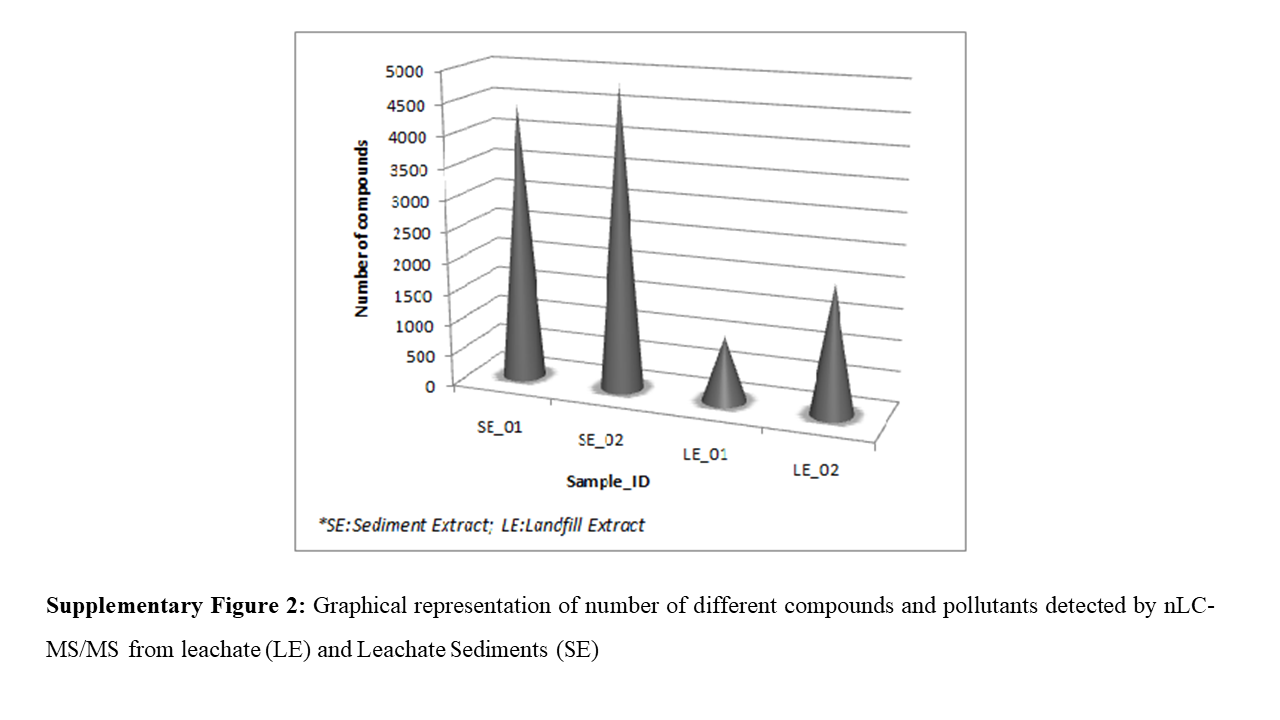

Supplement: Supplementary file 2 [file Image_2.tif]

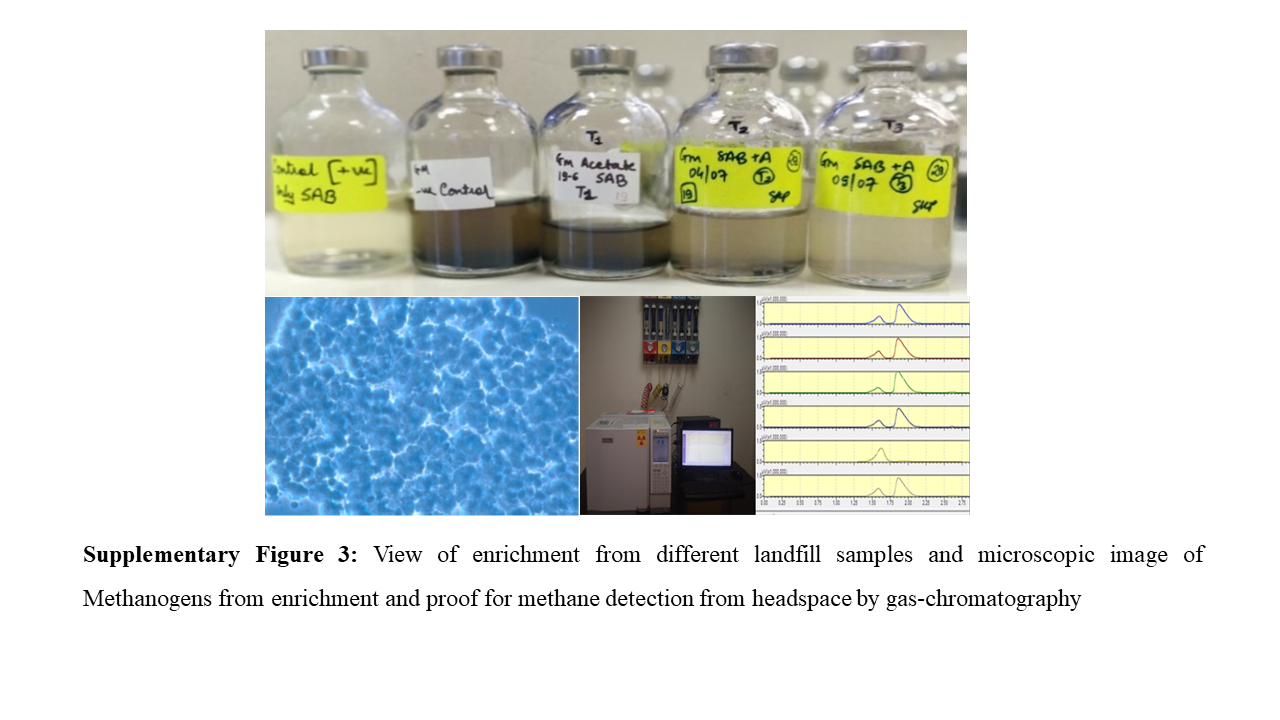

Supplement: Supplementary file 3 [file Image_3.tif]

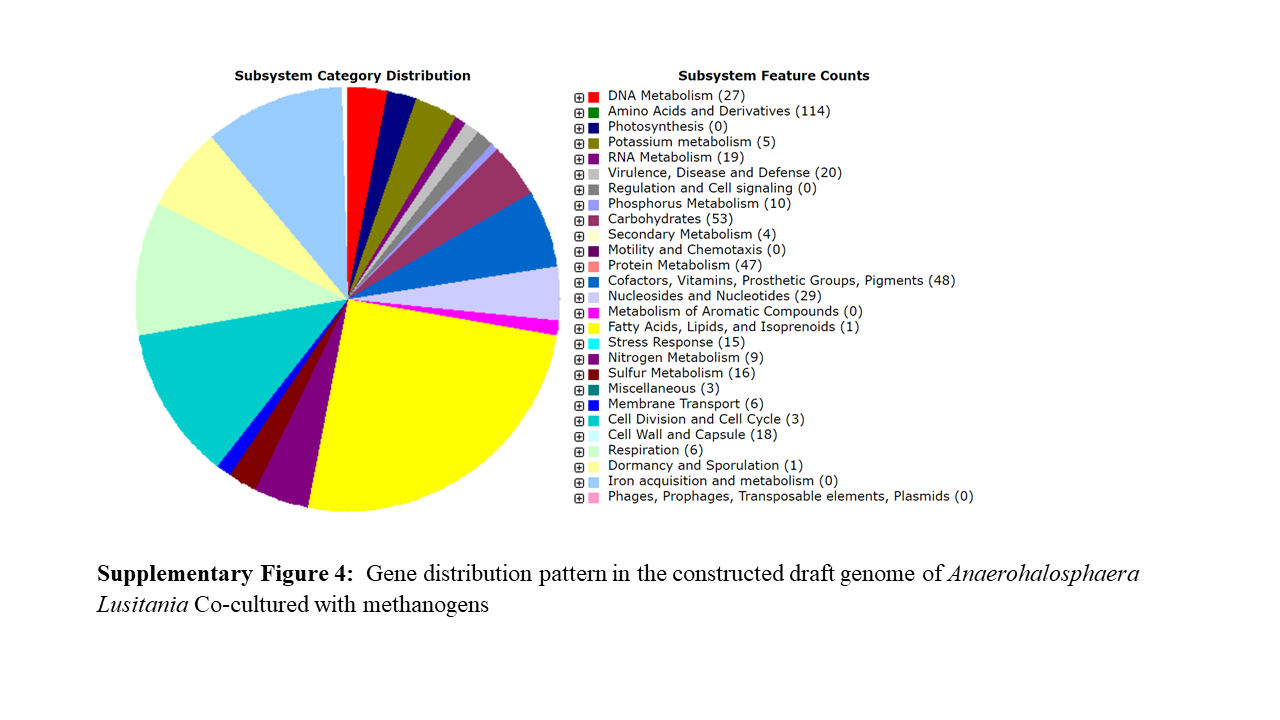

Supplement: Supplementary file 4 [file Image_4.tif]

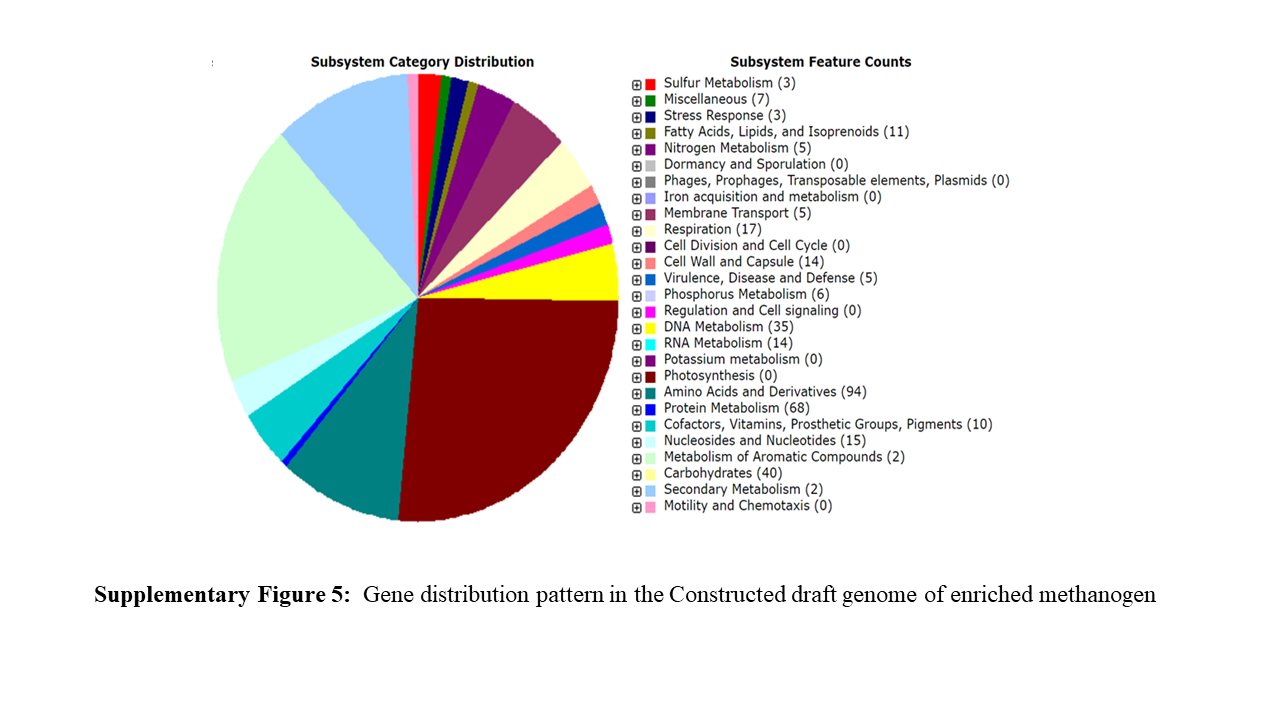

Supplement: Supplementary file 5 [file Image_5.tif]
